# The Genetic Programs Specifying Kolmer–Agduhr Interneurons

**DOI:** 10.3389/fnins.2020.577879

**Published:** 2020-10-09

**Authors:** Lixin Yang, Feifei Wang, Uwe Strähle

**Affiliations:** ^1^State Key Laboratory of Environmental Criteria and Risk Assessment, Chinese Research Academy of Environmental Sciences, Beijing, China; ^2^Center for Global Health, School of Public Health, Nanjing Medical University, Nanjing, China; ^3^Institute of Biological and Chemical Systems – Biological Information Processing, Karlsruhe Institute of Technology (KIT), Karlsruhe, Germany

**Keywords:** Kolmer–Agduhr cells, cerebrospinal fluid-contacting neurons, transcription factors, transcriptional regulatory network, GABAergic interneuron

## Abstract

Kolmer–Agduhr (KA) cells are a subgroup of interneurons positioned adjacent to the neurocoele with cilia on the apical surface protruding into the central canal of the spinal cord. Although KA cells were identified almost a century ago, their development and functions are only beginning to be unfolded. Recent studies have revealed the characteristics of KA cells in greater detail, including their spatial distribution, the timing of their differentiation, and their specification via extrinsic signaling and a unique combination of transcription factors in zebrafish and mouse. Cell lineage-tracing experiments have demonstrated that two subsets of KA cells, named KA’ and KA” cells, differentiate from motoneuronal progenitors and floor-plate precursors, respectively, in both zebrafish and mouse. Although KA’ and KA” cells originate from different progenitors/precursors, they each share a common set of transcription factors. Intriguingly, the combination of transcription factors that promote the acquisition of KA’ cell characteristics differs from those that promote a KA” cell identity. In addition, KA’ and KA” cells exhibit separable neuronal targets and differential responses to bending of the spinal cord. In this review, we summarize what is currently known about the genetic programs defining the identities of KA’ and KA” cell identities. We then discuss how these two subgroups of KA cells are genetically specified.

## Introduction

Kolmer–Agduhr (KA) cells are a group of cerebrospinal fluid (CSF)-contacting neurons (CSF-cNs). The term KA cell was first proposed by N. Dale et al. in 1987 (Dale et al., 1987b) to name a class of neurons that lie in the ventrolateral spinal cord and contact the cerebrospinal fluid in frog embryos (Roberts and Clarke, 1982); even earlier observations of cells with KA cell morphologies were made by Kolmer and Agduhr, who observed and described them in the spinal cords of most classes of vertebrates (Kolmer, 1921, 1925, 1931; Agduhr, 1922; Vigh-Teichmann and Vigh, 1983). Using antibodies against the neurotransmitter γ-aminobutyric acid (GABA) and an enzyme glutamic acid decarboxylase (GAD), numerous studies have reported the anatomy of KA cells in greater detail, including their axonal projection patterns, their appearance during development, and their distribution and organization in frogs (Dale et al., 1987a, b; Binor and Heathcote, 2001) and zebrafish (Bernhardt et al., 1992). For example, in frog (*Xenopus laevis*) embryos, KA cell have a pear-shaped soma (Roberts and Clarke, 1982). These GABA-positive KA cells distribute in the ventral part of the spinal cord in two orderly rows adjacent to the neurocoele (Dale et al., 1987b). There are numerous microvilli and one or two cilia on the apical surface of KA cells that project into the central canal of the spinal cord (Roberts and Clarke, 1982; Binor and Heathcote, 2001). Differentiated KA cells first appear at stage 25, and then one cell is continuously generated every 12 min on each side of the spinal cord (Dale et al., 1987b).

According to the location and origin of KA cells in zebrafish, two subsets of KA cells termed KA’ and KA” have been distinguished. KA” cells are distributed in the lateral floor plate (LFP), while the relatively dorsal KA’ cells localize in the motoneuron progenitor (pMN) domain (Park, 2004; Shin et al., 2007; Yang et al., 2010). Cell fate-mapping experiments showed that all KA’ cells are derived from Olig2+ precursors in the pMN domain (Park, 2004), while KA” cells differentiate from *nkx2.2a+*/*nkx2.2b+*/*nkx2.9+* progenitors in the lateral floor plate (LFP). Most KA cells are born around 16.5 h postfertilization (hpf) in zebrafish (Schäfer et al., 2007; Yang et al., 2010; Huang et al., 2012).

Similar subsets of KA cells are observed in the mouse spinal cord, where these cells are named CSF-cN’ and CSF-cN” (Petracca et al., 2016). CSF-cN’ cells are derived from *Nkx6+/Pax6+* progenitors positioned in the p2 neural progenitor domain and in the dorsal part of the oligodendrogenic (pOL) domain. In contrast, CSF-cN” cells originate from *Nkx2.2+/Foxa2+* precursors in the boundary between the p3 neural progenitor domain and the floor plate. Most CSF-cN cells are born around embryonic days 13–14 (E13–E14) (Petracca et al., 2016).

Neurons with somata that have similar characteristics to those of KA cells in terms of shape, position, and/or expression of GABA have been reported in the lancelet (Vígh et al., 2004), lamprey (Meléndez-Ferro et al., 2003; Jalalvand et al., 2014), dogfish (Sueiro et al., 2004), eel and trout (Roberts et al., 1995), newt (Harper and Roberts, 1993), and macaque (*Macaca fascicularis*) (Djenoune et al., 2014). Based on these comparative histological data, vertebrate KA cells are thought to be derived from an ancient epithelial neuron-like ectodermal cell (Vígh et al., 2004). This notion was further supported by a recent discovery of KA cells in the marine annelid (*Platynereis dumerilii*) (Vergara et al., 2017). Notably, compared with KA cells in the lamprey (Jalalvand et al., 2014) and zebrafish (Djenoune et al., 2017), mouse KA cells do not produce somatostatin (Petracca et al., 2016). There are thus important differences in the molecular identities of KA cells that have evolved over time.

The functions of KA cells have puzzled researchers for almost a century. According to the location and morphology of KA cells; the suggested physiological roles of these cells are mechanosensory or chemosensory (Kolmer, 1921; Agduhr, 1922; Vigh-Teichmann and Vigh, 1983). One recent *in vivo* experiment has demonstrated that KA cells have a direct mechanosensory function to sense CSF flow via polycystic kidney disease 2-like 1 (Pkd2l1) channels in the zebrafish spinal cord (Sternberg et al., 2018). In addition, there is evidence that KA cells may play a role as mechanoreceptors and chemoreceptors due to their expression of an acid-sensing ion channel (ASIC3) in lampreys (Jalalvand et al., 2016).

Knowledge of the shared expression of transcription factors and GABA neurotransmitter in KA’/CSF-cN’ and KA”/CSF-cN” cells allows one to ask how their common identities are genetically programmed. In this review, we will describe the gene expression patterns of KA/CSF-cN cells and summarize progress in the quest to understand how KA cell fates are specified. Finally, we will discuss possible future directions to provide additional details of the genetic programs that define a KA/CSF-cN cell fate.

## KA/CSF-cN Cells Are Gabaergic Interneurons

**FIGURE 1:**
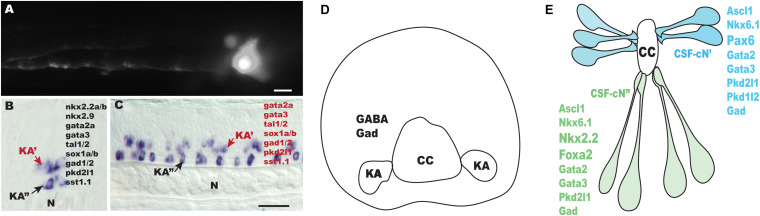
Outline of Kolmer–Agduhr (KA)/cerebrospinal fluid (CSF)-contacting neuron (cN) cells in different species. **(A)** sox1a+ KA” cell at 24-hpf-labeled transiently by injection of a GFP reporter cassette [TgBAC (*sox1a:eGFP*)] in zebrafish embryos at one-to-two cell stage. **(B,C)** Lateral view **(B)** and cross-section **(C)** of a 24-hpf zebrafish embryo hybridized with a *tal2* probe. **(D)** A scheme of a transverse section through the spinal cord of a frog embryo (stages 37–38). **(E)** A scheme of mouse CSF-cN cells (E14.5). N, notochord; CC, central canal. Scale bars: 25 μm in **(A)**; 50 μm in **(B,C)**.

Several characteristics of KA cells are provided in Figure 1 and Table 1. Cells with similar characteristics to those of KA cells, such as expressing the genes encoding Gad2 (formerly Gad65)/Gad1 (formerly Gad67) enzymes for the synthesis of GABA from glutamate, as well as releasing GABA have been identified in the lamprey (Jalalvand et al., 2014, 2016), dogfish (Sueiro et al., 2004), zebrafish (Bernhardt et al., 1992; Yang et al., 2010), frog (Dale et al., 1987b), mouse (Djenoune et al., 2014; Orts-Del’Immagine et al., 2014; Petracca et al., 2016), rat (Kútna et al., 2014), and macaque (*Macaca fascicularis*) spinal cords (Djenoune et al., 2014). Collectively, these findings demonstrate that KA cells are GABAergic interneurons that exhibit a long ascending ipsilateral axon. Of note, expressions of genes encoding somatostatin (sst), urotensin II-related peptides 1 (urp1) and 2 (urp2), and serotonin (5-hydroxytryptamine, 5-TH) are observed in lamprey (Jalalvand et al., 2014), dogfish (Sueiro et al., 2004) and zebrafish KA cells (Quan et al., 2015) (Djenoune et al., 2017), suggesting that KA/CSF-cN cells may play a role in exerting neuroendocrine activities.

**TABLE 1:** The characteristics of Kolmer–Agduhr (KA)/cerebrospinal fluid (CSF)-contacting neuron (cN) cells.

Organisms	KA cells	Birthdate of KA cells	Expressing genes	Proposed function in locomotion	References
Lamprey	KA	E10	GABA+, somatostatin+ (sst+)	These cells respond to both mechanical stimulation and to lowered pH, and may affect the locomotor-related sensory feedback	Meléndez-Ferro et al., 2003; Jalalvand et al., 2014; Jalalvand et al., 2016
Dogfish	CSF-cN’/KA’ arises from the lateral plate and locates in the most ventral region of the lateral walls.	Stages 25	GAD+, GABA+		Sueiro et al., 2004
	CSF-cN”/KA” arises from the floor plate	Stages 26	GAD+, GABA+, 5-TH+		
Zebrafish	KA’ originates from olig2+ P2 domain progenitors and locates more dorsally	10–15 hpf	Gata2+, gata3+, tal2+, tal1+, sox1a+, sox1b+, olig2+, gad65/67+, pkd2l1+, sst1.1+	Form projections onto V0v and commissural primary ascending (CoPA) sensory interneurons. Respond to lateral bending of the spinal cord. Project onto slow swimming circuits	Schäfer et al., 2007; Park, 2004; Yang et al., 2010; Huang et al., 2012; Yeo and Chitnis, 2007; Shin et al., 2007; Djenoune et al., 2014; Djenoune and Wyart, 2017; Quan et al., 2015; England et al., 2017; Higashijima et al., 2004
	KA” originates from progenitors of the lateral floor plate	Around 10 hpf	Pkd2l1+, nkx2.2a+, nkx2.2b+, nkx2.9+, gad65/67+, gata2+, gata3+, tal2+, tal1+, sox1a+, sox1b+, urp1+, urp2+, 5-HT+	Form projections onto caudal primary (CaP) motor neurons and commissural primary ascending (CoPA) sensory interneurons. Respond to longitudinal contractions. Trigger an activation of the locomotor network. Project onto fast swimming circuits	
Frog	KA	Stage 25	GAD+, GABA+		Roberts and Clarke, 1982; Roberts et al., 1987; Dale et al., 1987a
Chick	KA or SCF-cN	Stage 32	Pkd2l1		Petracca et al., 2016
Mouse	SCF-cN’ originates from P2 domain and dorsal half of pOL	E13–E14	Ascl1, Pax6+, Nkx6.1+, Gata2+, Sox2+, Pkd2l1+, Pkd1l2+, GAD+, vGAT+, β-III-tubulin+, Dcx+	Produce the repetitive spiking in 80% cells	Petracca et al., 2016; Djenoune et al., 2014; Kútna et al., 2014; Orts-Del’Immagine et al., 2014; Di Bella et al., 2019
	SCF-cN” originates from progenitors adjacent to the floor plate		Ascl1, Nkx6.1+, Nkx2.2+, Foxa2+, Sox2+, Pkd2l1+, Pkd1l2+, GAD+, vGAT+, β-III-tubulin+, Dcx+,	Produce a single spike	
Rat	SCF-cN positions in the lateral part of the central canal	E13	DCX+, GABA+, GAD65+		Kútna et al., 2014
	SCF-cN positions in the ventral part of the central canal	E12	DCX+, GABA+, GAD65+		
Macaque	CSF-cN cells		GAD65/67+, PKD2L1+		Djenoune et al., 2017

## Subsets of KA/CSF-cN Cells Are Differentiated From Different Developmental Origins

KA cells are subdivided into the KA”/CSF-cNs” ventral subgroup and KA’/CSF-cNs’ dorsal subgroup in zebrafish (Yang et al., 2010), dogfish (Sueiro et al., 2004), mouse (Petracca et al., 2016), and rat (Kútna et al., 2014). Considering the distinct locations of each of these KA cells subtypes, it is hypothesized that KA’ and KA” cells are generated from different developmental origins. Based on cell fate mapping and clonal analysis, indeed, KA’ cells are distinguished as a subgroup of interneurons expressing *olig2:EGFP+/GABA+* in zebrafish embryos, whereas KA” cells are found to be generated from LFP *nkx2.2+/nkx2.9+* progenitors. Evidence supports that KA’ cells are differentiated from *olig2+* progenitors. First, cell fate-mapping experiments have shown that in zebrafish, all KA’ cells are derived from the *olig2+* precursors in the pMN domain, which also produces motoneurons (Park, 2004). Second, morpholino knockdown of *olig2* abolishes cells expressing KA’ markers including *tal2* and *gad65/67* (Yang et al., 2010). In contrast, current evidence supports that KA” cells are differentiated from LFP *nkx2.2+/nkx2.9+* progenitors. Specifically, *nkx2.2+/nkx2.9+* progenitors divide both symmetrically and asymmetrically and form KA” cells in zebrafish embryos (Huang et al., 2012). In addition, morpholino knockdown of *nkx2.2a*, *nkx2.2b*, and *nkx2.9* completely eliminates KA” cells expressing the markers *gata2a*, *gata3*, *sox1a*, *sox1b*, *tal2*, and *gad65/67* in the LFP (Yang et al., 2010; Gerber et al., 2019). Furthermore, a subset of KA” cells expressing *tal2+/nkx2.2b+* differentiates into *sim1+/huC/D+* V3 interneurons, and thus, *tal2+/nkx2.2b+* cells are postulated to be p3 neural progenitor cells (Schäfer et al., 2007). This notion has been further supported by a recent report that shows that in *gata2a* mutants, KA” cells lose their identities, and that there is a concomitant increase in the number of cells expressing the V3-specific gene, *single-minded homolog 1a* (*sim1a)*, which encodes a leucine zipper/PAS transcription factor gene single-minded homolog 1a (Andrzejczuk et al., 2018).

To determine the developmental origins of KA cells in mouse, newly born *Pkd2l1*-expressing CSF-cN cells have been mapped in relation to the domains marked by transcription factors including Nkx6.1, Pax6, Nkx2.2, and Olig2. These experiments have shown that 70% of CSF-cN’s arise from the Nkx6.1+/Pax6+ progenitors located dorsal to Olig2+ ventricular cells, (which marks the p2 neural progenitor domain), whereas the other 30% of these cells are differentiated from the dorsal half of the Olig2+ pOL domain; in contrast, CSF-cN”s were found to originate from the Nkx2.2+/Foxa2+ cells positioned in the floor plate (Petracca et al., 2016). Taken together, the current evidence supports that at least two subgroups of KA/CSF-cN cells develop from distinct progenitors in zebrafish and mouse. Of note, regardless of the different origins of CSF-cN’ and CSF-cN” cells in Ascl1-deficient mice, both of these CSF-cN subtypes fail to differentiate, and CSF-cN precursors are instead converted into non-neuronal ependymocytes (Di Bella et al., 2019), suggesting that Ascl1 may play a role as a selector for controlling the fate of CSF-cN cells and ependymocytes in mouse.

## Transcription Factors Driving the Identities of KA/CSF-cN Cells

To better understand how KA/CSF-cN cells are generated, several studies have made progress by investigating the genetic programs that regulate KA cell development. Currently, at least 10 transcription factors have been identified to be involved in specifying KA/CSF-cN cells in zebrafish and/or mouse.

*Nkx2.2* and *nkx2.9* each contain highly conserved homeobox and NK2-specific domains and belong to the family of class II transcription factors. Zebrafish have two *nkx2.2* genes, namely, *nkx2.2a* and *nkx2.2b* (Schäfer et al., 2005). The spatial expressions of *nkx2.2a*, *nkx2.2b*, and *nkx2.9* are restricted to the LFP (Schäfer et al., 2005) (Yang et al., 2010). In the zebrafish LFP, there are at least three different cell groups positioned along the anteroposterior axis. One of these subgroups has been identified as KA” cells and expresses *nkx2.2a*, *nkx2.2b*, *nkx2.9*, and *tal2*. The functions of *Nkx2.2a*, *Nkx2.2b*, and *Nkx2.9* are necessary for guiding the identity of *gad65/67* expressing KA” cells in a functionally redundant manner (Yang et al., 2010). The second subgroup of cells expressing *nkx2.2a* and *nkx2.9* are thought to be undifferentiated LFP progenitor cells. Differentiated KA” cells downregulate the expressions of *nkx2.2a* and *nkx2.9* (Huang et al., 2012). The third subgroup of *tal2+/nkx2.2b+* cells differentiates into *sim1+* V3 postmitotic interneurons (Schäfer et al., 2007). Morpholino knockdown experiments have revealed that *nkx2.2a* and *nkx2.2b* are required for the formation of LFP cells, but are not essential for defining *tal2+*/*nkx2.2b+* cells. Furthermore, cells expressing *foxa2* and *nkx2.2b* represent the non-neuronal floor plate cells and proliferate during early neurogenesis (Schäfer et al., 2007).

In mouse, CSF-cN” cells express Nkx2.2 and Foxa2; however, they do not express Lmx1b, a marker of the non-neurogenic floor plate, or Pax6, suggesting that Pkd2l1+ CSF-cN” neurons developed from the boundary between the p3 ventricular zone and the floor plate (Petracca et al., 2016). Nkx2.2 is expressed in CSF-cN” cells, but it is not essential for the differentiation of Pkd2l1+ CSF-cN” cells because no difference is observed in the number of Pkd2l1+ KA” cells in *Nkx2.2* mutants compared to that in controls (Petracca et al., 2016). One possible explanation for this result is that there is functional redundancy of Nkx2.2 and Nkx2.9 for specification of Pkd2l1+ CSF-cN” cells, as found in zebrafish. However, whether these different cell types exist in the mouse LFP remains unclear.

There are two *nkx6* homologs in zebrafish, named *nkx6.1* and *nkx6.2*. They are each expressed in the ventral spinal cord, including within the floor plate and pMN domain. In the absence of Nkx6.1 and Nkx6.2 proteins, middle primary motoneurons (MiPs) develop a hybrid phenotype consisting of morphological characteristics of both motoneurons and interneurons; however, the number of GABA-positive cells produced from the pMN domain and LFP do not change (Cheesman, 2004; Hutchinson et al., 2007). In mouse, Nkx6.1 is expressed by both CSF-cN’ and CSF-cN” cell progenitors. CSF-cN’ and CSF-cN” cells are derived from Nkx6.1+/Pax6+ and Nkx6.1+/Nkx2.2+/Foxa2+ progenitors, respectively, but the functions of Nkx6.1 in the specification of CSF-cN subtypes have not yet been reported.

*Gata2a* and *gata3* belong to the C4 zinc-finger family and are expressed by the V2b, V2s, KA”, and KA’ cells in zebrafish (Batista et al., 2008; Gerber et al., 2019; Yang et al., 2010). Morpholino knockdown of *gata3* eliminates KA’ cell formation (Yang et al., 2010). Consistent with this finding, several KA’ markers, including *tal2*, *gad65/67*, *pkd2l1*, and *sst1.1* are completely abolished in *gata3* mutants (Andrzejczuk et al., 2018), suggesting that Gata3 is required for specifying KA’ cells. While knockdown of *gata2a* dramatically reduces *gad65/67*-expressing KA” cells, the expressions of several KA” markers, including *gata3*, *tal1*, *sox1a*, *gad65/67*, *pkd2l1*, and *urp1* are eliminated in *gata2a* mutants (Yang et al., 2010) (Andrzejczuk et al., 2018). These data suggest that *gata2a* and *gata3* denote distinct regulatory networks for specifying KA” and KA’ cells, respectively, despite *gata2a* and *gata3* being expressed in both KA” and KA’ cells. In mouse, CSF-cN cells are identified as late born neurons appearing at E14.5 and express Gata2, Gata3, Pkd2l1, and Pkd1l2; however, the functions of Gata3 and Gata2 in CSF-cN cells have not yet been reported (Petracca et al., 2016).

*Olig2*, *a* basic helix–loop–helix (bHLH) transcription factor, plays a pivotal role in oligodendrocytic and motoneuronal differentiation. *Olig2* is expressed in proliferative ventral neuronal precursors, primary motoneurons, and oligodendrocytic progenitors in zebrafish (Park, 2004). Cell tracking experiments have suggested that all KA’ cells are differentiated from the Olig2+ progenitors in zebrafish and that the function of Olig2 is required for the production of KA’ cells from progenitors in the pMN domain (Park, 2004; Yang et al., 2010). In mouse, nearly 70% of CSF-cN’ cells are produced from progenitors with a p2 identity, whereas only 30% originate from the Olig2+ cells. One possible explanation is that Olig2 may be transiently expressed by p2 progenitors, but that CSF-cN’ cells differentiate several days later. Hence, it remains to be determined whether Olig2 plays a role in the development of mouse CSF-cN’ cells.

*Tal1* and *tal2* belong to the family of bHLH transcription factors. Both of *tal1* and *tal2* share 50% identical amino acids and are expressed by KA,” KA,’ and V2b cells in zebrafish (Andrzejczuk et al., 2018; Pinheiro et al., 2004; Yang et al., 2010). Genetic inhibition of *tal1* in homozygous *tal1* mutants abolishes the expressions of *gata3*, *gata2a*, *tal2*, *sox1a*, *sox1b*, *gad65/67*, *pkd2l1*, and *sst1.1* in KA’ cells, whereas knockdown of *tal2* causes a reduction in the KA” markers, *gad65/67* expression (Andrzejczuk et al., 2018; Yang et al., 2010), even though *tal1* and *tal2* are expressed in both KA” and KA’ cells. This suggests that *tal1* and *tal2* may combine with different transcription factors and form a distinct regulatory network to differentially specify KA” and KA’ cells.

*Sox1a* and *sox1b* belong to group B of the *Sox* gene family and share 86% amino acid sequence identity. *Sox1a* and *sox1b* are expressed by KA,” KA,’ V2b, and V2s interneurons in zebrafish (Andrzejczuk et al., 2018) (Gerber et al., 2019). Knockdown of *sox1a* and *sox1b* results in a significant increase in the expression levels of V2b markers, including *tal2*, *gata2a*, *gata3*, and *gad65/67* in the V2 domain, whereas markers for KA cells are unaffected. In agreement with this finding, *sox1a* and *sox1b* mutants only affect the expression levels of V2b markers (Gerber et al., 2019), indicating that *sox1a* and *sox1b* are expressed by KA cells, but that they are dispensable for KA cell specification. In mouse, Sox1, the ortholog of zebrafish *sox1a* and *sox1b*, is expressed in the ventricular progenitor zone in the spinal cord and in V2c interneurons. In the absence of Sox1, V2c interneurons become reprogrammed toward the V2b cell fate, suggesting that Sox1 is essential for the specification of the V2c interneuronal fate (Panayi et al., 2010). However, it remains to be determined whether the function of Sox1 plays a role in specifying CSF-cN cells in mouse.

*Ascl1*, a bHLH transcription factor, is expressed by the CSF-cN lineage and plays an important role in CSF-cN development (Di Bella et al., 2019). In mice lacking *Ascl1*, expressions of Gata2, Gata3, Pkd2l1, and Pkd1l2 in CSF-cN cells are abolished, and prospective CSF-cN progenitors instead adopt the morphology of central canal ependymocytes. Remarkably, simultaneous knockdown of *ascl1a* and *ascl1b* in zebrafish results in a reduction (*∼*40%) of *pkd2l1+* KA cells without eliminating either KA’ or KA” cells, suggesting that the activity of Ascl1 in defining the identities of KA/CSF-cN cell identity in zebrafish differs from that in mouse, the latter of which is fully dependent on Ascl1.

*Pax6* is a member of transcription factors containing a paired box. In mouse, Pax6 is expressed by most dorsal subgroups of Pkd2l1+ CSF-cN’ cells, and the expression of Pax6 is sharply downregulated during CSF-cN’ neurogenesis (Petracca et al., 2016). In the absence of Pax6, the number of Pkd2l1+ CSF-cN’ cells is almost entirely diminished, whereas the number of CSF-cN” cells positive for Pkd2l1, Nkx2.2, and Foxa2 remain unchanged, suggesting that Pax6 is only required for specifying Pkd2l1-expressing CSF-cN’ cells (Petracca et al., 2016). Despite these findings in mouse, it remains unclear whether Pax6 plays a similar role in specifying KA’ cells in zebrafish.

## Transcription Factors That Are Not Expressed in KA Cells but Are Involved in Their Specifying

*Islet1* is a member of the subfamily of LIM homeobox genes, a class of genes that control cell-fate programs in vertebrates. Zebrafish *islet1* is expressed by motoneurons and plays a prominent role in motoneuronal development (Hutchinson, 2006). Dorsally projecting MiPs express *islet1*. KA’ cells do not express *islet1*; however, knockdown of *islet1* significantly increases the number of GABA-expressing ventrolateral descending (VeLD) interneurons and KA’ cells, without disrupting the number of GABA-expressing cells at the location in which KA” cells are normally located (Hutchinson, 2006). Consistent with this finding, misexpression of Islet1 significantly reduces the number of GABA-expressing VeLD (V2b) interneurons and KA’ cells, whereas the number of cells in the KA” position is not changed compared with that in the control (Hutchinson, 2006). A possible explanation for this phenomenon is that zebrafish Iselt1 may function to promote the formation of primary motoneuron formation and mediate a switch between motoneuronal and interneuronal fates in the pMN domain. Although this study only determined the number of GABA-expressing KA and VeLD cells in the absence or misexpression of Iselt1, several other lines of evidence support the idea that KA’ cells, but not the VeLD interneurons, may be the target of *iselt1*-mediated patterning. First, KA’ cells are derived from Olig2+ progenitors positioned in the pMN domain, and the activity of Olig2 is required for KA’ cell specification (Park, 2004; Yang et al., 2010). Second, the effects of misexpression of *islet1* is limited to a subset of interneurons produced from the pMN domain (Hutchinson, 2006). Third, VeLD/V2b interneurons express *lhx3* but not *islet1* (Appel, 1995). Fourth, the number of V2b is unchanged in the absence of Olig2, whereas a lack of Olig2 abolishes nearly all primary motoneurons expressing *islet2*, as well as nearly all KA’ cells (Park, 2004; Yang et al., 2010).

*Lhx3* and *lhx4* genes belong to the family of LIM homeodomain transcription factor and play pivotal roles in motoneuronal and interneuronal differentiation. In the absence of *lhx3* and *lhx4*, primary motoneurons develop a hybrid identity in which *islet*-expressing neurons coexpress GABA and *gad*, and form ipsilateral ascending axons, a characteristic property of the KA’ cells (Seredick et al., 2014). Evidence supports the idea that Lhx3 and Lhx4 may regulate Notch signaling, which in turn promotes the expression of *gad* in primary motoneurons. Forced-expression experiments have demonstrated that Lhx3 promotes the specification of circumferential descending (CiD) interneurons, (also known as V2a interneurons) at the expense of KA’ cells. Although *lhx3* and *lhx4* are not expressed in KA’ cells, Lhx proteins can regulate the expression levels of *gad* and GABA in primary motoneurons and influence axonal projections to acquire the phenotype of ipsilaterally ascending axons (Seredick et al., 2014).

## Potential Markers of KA/CSF-cN Cells

### Pkd1l2a and Pkd2l1

The polycystic kidney disease (PKD) gene family encodes transmembrane proteins that share a conserved polycystin-cation-channel domain. Several lines of evidence support that genes encoding PKD 1-like 2a (*pkd1l2a*) and *pkd2l1* are expressed by all KA” and KA’ cells in zebrafish embryos (Djenoune et al., 2014; England et al., 2017), while *Pkd2l1* is also expressed in mouse and macaque KA cells (Djenoune et al., 2014). Approximately 15% of PKD2L1+ KA cells are GABA/GAD67 negative in the adult mouse spinal cord. PKD2L1+ KA cells are not serotonergic (5-HT) or catecholaminergic [marked by tyrosine hydroxylase (TH) expression] (Djenoune et al., 2014). A potential explanation for this discrepancy may be due to differences in embryonic and adult tissues. *In vivo* experiments suggest that *pkd2l1* is required for KA cells to detect CSF flow in zebrafish embryos; however, Pkd2l1 is not required for KA cell differentiation (Sternberg et al., 2018).

## KA’/CSF-cN’ and KA”/CSF-cN” Cells Share Common Transcription Factors but Differ in Terms of Their Regulatory Networks

We and others have shown that KA’ and KA” cells share a group of transcription factors including *gata2a*, *gata3*, *tal1*, *tal2*, *sox1a*, and *sox1b* in zebrafish embryos (Yang et al., 2010; Andrzejczuk et al., 2018; Gerber et al., 2019). However, the genetic programs regulating KA’ and KA” development are distinct from one another. Morpholino knockdown analyses have indicated that *gata3* is required for KA,’ but not KA” cell specification, whereas *gata2a* and *tal2* are indispensable for specification of KA” but not KA’ cells (Yang et al., 2010). Consistent with these results, analyses of *tal1*, *gata2a*, and *gata3* mutant have demonstrated that Gata2a is required for specifying KA” cell identity, and that Gata3 and Tal1 are required for defining KA’ cell fate (Andrzejczuk et al., 2018). Deficiency of *gata2a* results in a loss of cells in the LFP (where KA” cells are generated) that expresses *gata3*, *tal2*, *tal1*, *sox1a*, *sox1b*, *gad65/67*, *urp1*, and *pkd2l1*, but not a loss of such cells in the dorsal spinal cord where KA’ cells normally form (Yang et al., 2010; Andrzejczuk et al., 2018; Gerber et al., 2019) (Yang et al., unpublished observations). In addition, a significant increase in the number of *slc17a6a/b* and *sim1a*-expressing cells is observed in *gata2a* mutant (Andrzejczuk et al., 2018), suggesting that at least some KA” cells shift to become V3 interneurons or adopt a hybrid V3/KA” fate in the absence of *gata2a*. Further investigation has revealed that knockdown of *tal2* eliminates the expression of *gad65/67* in KA” cells, whereas the expressions of *gata2a* and *gata3* in KA” cells are unchanged. Taken together, current evidence suggests that *gata2a* acts upstream of *tal2* and *sox1a* in KA” cells, which in turn drive the expressions of *gad65/67*, *urp1*, and *pkd2l1* in KA” cells.

In the absence of Gata3 protein, KA’ cells that express *gata2a*, *tal1*, *tal2*, *sox1a*, *sox1b*, *gad65/67*, *sst1.1*, and *pkd2l1* are abolished, whereas there is no change in the number of KA” cells expressing *gata2a*, *tal1*, *tal2*, *sox1a*, *sox1b*, *gad65/67*, *sst1.1*, and *pkd2l1* (Yang et al., 2010; Andrzejczuk et al., 2018; Gerber et al., 2019) (Yang et al. unpublished observations). Similarly, in *tal1* mutants, expressions of *gata2a*, *tal2*, *sox1b*, *gad65/67*, *sst1.1*, and *pkd2l1* in KA’ cells are completely abolished, and *gata3* and *sox1a-*expressing KA’ cells are dramatically reduced. In contrast, there is no effect on the number of KA” cells (Andrzejczuk et al., 2018). Furthermore, an increase in the number of phosphor-histone H3-positive/*olig2*-positive cells positioned in the pMN domain (where KA’ cells are generated) is observed in both *gata3* and *tal1* mutants, suggesting that loss of the function of Gata3 and/or Tal1 may promote cells to become mitotically active precursors, which in turn block/delay KA’ cell differentiation. Similarly, Gata2/3 are expressed in mouse CSF-cN’ and CSF-cN” cells, although expressions of Tal1 and Tal2 were not examined in this study (Petracca et al., 2016). Gene function analysis demonstrates that Pax6 is exclusively required for the production of CSF-cN’ cells from progenitors in the p2-pOL domain. In contrast, Nkx2.2 is dispensable for the production of CSF-cN” cells despite CSF-cN” cells expressing Nkx2.2. Despite these recent findings, further studies are needed to elucidate the functions of Gata2, Gata3, Tal1, and Tal2 in regulating the CSF-cN cell differentiation in mouse.

## Specifications of KA’/CSF-cN’ and KA”/CSF-cN” Cells Are Differently Regulated by Hedgehog and Delta-Notch Signaling

Hedgehog signaling plays a pivotal role in defining the KA” cell fate in a concentration- and duration-dependent manner (Strähle et al., 2004; Schäfer et al., 2007; Huang et al., 2012). Loss of sonic hedgehog (Shh) signaling in homozygous mutants of the ligand Shh (sonic-you, *syu*), the signal transducer smoothened (slow-muscle-omitted, *smo*), and the transcription factors Gli1 (detour, *dtr*) and Gli2 (you-too, *yot*) completely eliminates expressions of several markers, namely, *nkx2.2a*, *nkx2.2b*, *nkx2.9*, and *tal2* in the LFP and in KA” cells (Yang et al., 2010) and Yang et al., unpublished observations) (Schäfer et al., 2007). In contrast, the expression of *tal2* in KA” cells is normal in heterozygous *dtr* and *yot* mutants (Schäfer et al., 2007), suggesting that compared with those in *nkx2.2b+/foxa2+* LFP cells, relatively lower levels of hedgehog activity are required for forming KA” cells (*Nkx2.2b+/Tal2+*) and *Sim1*-positive V3 interneurons in zebrafish (Schäfer et al., 2007). In agreement with this, the LFP progenitors remain responsive to hedgehog, whereas differentiated KA” cells lose their responses (Huang et al., 2012). Further evidence indicates that forced expression of Gli1 reduces the number of KA” cells and increases in *nkx2.9*-expressing LFP cells, suggesting that termination of hedgehog signaling is essential for KA” cell differentiation (Huang et al., 2012). In addition, activation of hedgehog signaling by ectopic expression of Shh or the dominant-negative form of PKA mRNA induces numerous *tal2*-expressing KA” cells, as well as dorsally located KA’ cells (Huang et al., 2012). Intriguingly, expression of *tal2* in more dorsally located cells, which might represent KA’ cells and V2b interneurons, is unaffected in the absence of Gli2 (Schäfer et al., 2007). This phenomenon appears to hold true in embryos incubated in cyclopamine from the shield stage to the 22 somite stage, in which *tal2*-positive KA” cells are completely eliminated, whereas the *tal2*-positive KA’ cells are not, and V2b interneurons also likely exist (Schäfer et al., 2007). These results suggest that hedgehog signaling may play differential roles in specifying KA” and KA’ cells.

Comparative studies suggest that the functions of hedgehog signaling in mouse differ from those in zebrafish (England et al., 2011). Hedgehog signaling is required to induce both V3 interneurons in the p3 domain and some motoneurons in the pMN domain. Loss of Shh signaling in mouse results in severely decreased numbers of V1 and V0v cells, in which case only a few V2 interneurons form, and there is a complete elimination of motoneurons. Additionally, a lack of hedgehog signaling in zebrafish embryos results in most V3 domain cells not forming and motoneurons being dramatically reduced (England et al., 2011). However, it is unclear whether hedgehog signaling plays a role in defining the CSF-cN identities in mouse.

Notch signaling has been implicated in KA cell development (Schäfer et al., 2007; Shin et al., 2007; Yeo and Chitnis, 2007; Huang et al., 2012). Absence of Notch signaling in the zebrafish mutant, *mindbomb* (*mib*), which encodes an E3 ubiquitin ligase and is necessary for efficient Notch signaling (Itoh et al., 2003), results in loss of both LFP and KA” cells (Schäfer et al., 2007; Yeo and Chitnis, 2007). In addition, early blocking of Notch signaling by expressing a dominant-negative form of *Xenopus suppressor* of *Hairless* [Su(H)] or inhibitors at 7 hpf leads to a reduction in the number of KA” cells, as that observed in the *mib* mutant (Schäfer et al., 2007; Yeo and Chitnis, 2007; Huang et al., 2012). Inhibition of Notch signaling from 10 to 25 hpf results in a significant increase in the number of *tal2*-expressing KA” cells at the expense of *nkx2.9*-expressing FLP cells (Huang et al., 2012). Conversely, activation of Notch signaling by the induced Notch intracellular domain (NICD) at 10 hpf almost completely eliminates *tal2*-expressing KA” cells, but increases the LFP cells expressing *nkx2.9* (Huang et al., 2012). In contrast, blocking Notch signaling at 17 hpf does not affect the number of KA” cells (Yeo and Chitnis, 2007). Furthermore, knockdown of *Jagged2*, a ligand of Notch receptors, causes a significant increase in the number of KA” cells and secondary motor neurons (SMNs), as well as a significant decrease in the rate of cell division. These data suggest that Jagged2-mediated signaling is not only required to maintain a group of dividing precursors, but that it also plays a role in regulating the number of KA” cells. Notch signaling also plays a pivotal role in specifying KA’ cells. In the absence of Notch signaling, primary motoneurons are formed at the expense of KA’ cells. In contrast, an excess of Notch signaling induces KA’ cell formation at the expense of PMNs in zebrafish, suggesting that Notch signaling promotes KA’ cell identity and inhibits primary motoneuronal fate (Shin et al., 2007). These lines of evidence support that Notch signaling plays an essential role in KA cell differentiation. Hence, specification of KA” cells initially depends on the activation and then the attenuation of both Notch and hedgehog signaling (Huang et al., 2012).

## The Transcriptional Regulatory Logic That Drives KA/CSF-cN Identity

**FIGURE 2:**
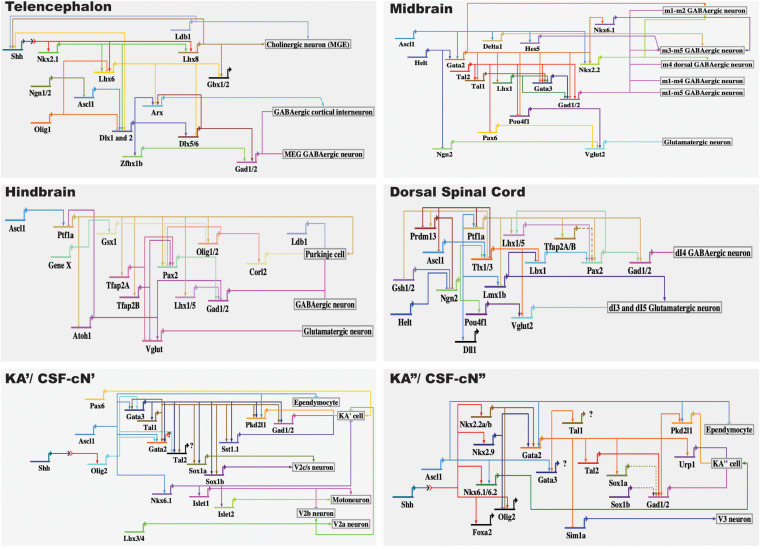
Gene regulatory network leading to differentiation of GABAergic interneurons. Of note, arrows do not necessarily reflect direct interactions between genes or proteins. The BioTapestry was used for building the GRN (http://www.biotapestry.org/).

Based on findings by our lab and other research groups (Park, 2004; Yeo and Chitnis, 2007; Yang et al., 2010; Petracca et al., 2016; Andrzejczuk et al., 2018; Di Bella et al., 2019; Gerber et al., 2019), here, we summarize the regulatory network guiding the KA/CSF-cN differentiation and identity (see Figure 2). Considering that KA/CSF-cN cells are GABAergic neurons, we summarize the transcriptional regulatory network guiding GABAergic neuronal identity in the mouse telencephalon, midbrain, hindbrain, and dorsal spinal cord (Figure 2). A line of evidence supports that the genetic program guiding GABAergic fate is likely dependent on multiple transcription factors in different regions, rather than by universal regulators that govern differentiation of all GABAergic neurons (Achim et al., 2014; Hobert and Kratsios, 2019). Furthermore, there is conceivable evidence supporting that differences in the transcription regulatory networks controlling generation of the diversity of GABAergic neurons may depend on the respective selector gene being either selectively antagonized by a repressor and/or assisted by region-specific cofactors (Hobert and Kratsios, 2019). Nevertheless, transcription factors including proneural genes (e.g., *Ascl1*, *Helt*) and postmitotic subtype selector genes (e.g., *Gata2*, *Gata3*, *Tal1*, and *Tal2*) appear to be repeatedly employed for driving GABAergic identity in mouse. In particular, functions of PTF1A and GATA2/TAL1 have been demonstrated to play a role as a GABAergic, rather than glutamatergic selectors in the dorsal and ventral spinal cord, respectively. In addition, *Dlx1/2*, *Gata2*, and *Gata2*/*Tal2* have been suggested to play roles as selectors for GABAergic neuronal identity in the mouse telencephalon, diencephalon, and midbrain, respectively (Achim et al., 2014; Figure 2).

KA’ and KA” cells share a class of the transcription factors, including *gata2*, *gata3*, *tal1*, *tal2*, *sox1a*, and *sox1b*, despite these cellular subtypes having different developmental origins. This is in agreement with observations that similar neurons, such as dopaminergic and GABAergic neuronal classes with distinct lineages, appear to be specified by the same terminal selector type transcription factors in *C. elegans* (Gendrel et al., 2016). We surmise here, as suggested via terminal selectors elucidated previously (Hobert, 2016) that *gata3/tal1* and *gata2/tal2* may serve as terminal selectors controlling KA’ and KA” differentiation, respectively, by combining cis-regulatory motifs associated with *gad1/2* and/or *pkd2l1/pkd1l2* genes in zebrafish. *Gata3* and *tal1* are expressed in KA’ and KA” cells; however, genetic removal of *gata3* and *tal1* only leads to a failure of KA’ to be differentiated from precursors. Similarly, *gata2/tal2* are expressed in both KA’ and KA” cells, but genetic removal of *gata2* and *tal2* only leads to a failure of KA” cells to acquire a GABAergic identity (Yang et al., 2010; Andrzejczuk et al., 2018). This is consistent with the function of *Gata2* in specifying GABAergic identity in the mouse midbrain and in rhombomere 1. *Gata2* is required for GABAergic neuronal differentiation in the midbrain. However, expressions of the GABAergic marker genes, *Gata3* and *Gad1*, in GABAergic precursors of rhombomere 1 are not altered in the *Gata2* mutants (Kala et al., 2009). The most likely explanation for these observations is that the differences in the cooperation of *gata3/tal1* and *gata2/tal2* for specifying the KA’ and KA” cell identity may be related to the different lineages of KA’ and KA” cells. *Gata3* may cooperate with the cofactor *tal1* to define KA’ identity. In line with this hypothesis, deficiency of *tal1* phenocopies the characteristics of *gata3* mutants, including the elimination of KA’ cells expressing *gata2a*, *gata3*, *tal2*, and *gad1/2* (Andrzejczuk et al., 2018). Similar to findings in *tal1* mutants, in the absence of *gata3*, expressions of *gata2a*, *tal1*, *tal2*, and *gad1/2* are abolished in KA’ cells. In addition, deletion of *tal1* phenocopies loss of expression of *Tg(-8.1gata1-EGFP)* in the V2b region observed in *gata2a*/*gata3* double mutants, suggesting that *gata2a* and *gata3* may cooperate with their cofactor, *tal1*, as a functional complex for specifying V2b interneurons in zebrafish (Andrzejczuk et al., 2018).

In mouse, both CSF-cN’ cells and V2b interneurons share the expressions of *Gata2* and *Gata3*. However, evidence supports that CSF-cN’ cells are different from early born GATA2 and GATA3-expressing V2b interneurons. In contrast with the finding that *Foxn4* is essential for V2b interneuronal specification, differentiation of CSF-cN’ cells is unchanged in the *Foxn4* mutants. Moreover, the activity of PAX6 is indispensable for CSF-cN’ specification, but V2b interneurons are not affected in Pax6 mutant mice (Petracca et al., 2016).

## Differences Between KA/CSF-cN Differentiation in Mouse and Zebrafish

Since the underlying mechanisms and signaling controlling the formations of the medial floor plate and LFP are different in mouse and zebrafish (Strähle et al., 2004), the genetic programs defining the identity of KA/CSF-cN cells may differ in these two vertebrate species. Indeed, in Ascl1-deficient mouse, CSF-cN cells fail to initiate differentiation, and the precursors are converted into ependymal cells. In contrast, in the knockdown of *ascl1a* and *ascl1b*, KA cells are still formed, despite a decrease in the numbers of KAs observed in zebrafish (Di Bella et al., 2019). Whether *Ascl1* plays a similar role in differentiation of early born KA/CSF-cN cells in *Xenopus* and lamprey as that does in zebrafish remains to be elucidated. In addition, observations have shown that CSF-cN cells are differentiated only after a neurogenic-to-gliogenic switch of spinal precursors in mice, rats, and chicks (Petracca et al., 2016) (Kútna et al., 2014) (Di Bella et al., 2019). In contrast to findings in mouse, in zebrafish and *Xenopus*, KA cells are produced simultaneously with primary motoneurons and other interneurons.

## Discussion

Although it is currently known that *gata3/tal1* and *gata2/tal2* drive KA’ and KA” identities, respectively, in the zebrafish spinal cord, it remains unclear how *gata3/tal1* and *gata2/tal2* genes are selected and functionally define these two groups of KA cells despite all of these genes being expressed in both KA’ and KA” cells. In addition, at least some KA” cells change from a GABAergic identity to a glutamatergic V3 interneuronal identity or acquire a hybrid V3/KA” identity in *gata2a* mutant zebrafish. Furthermore, in the absence of both *gata3* and *tal1*, an increase in the numbers of phosphor-histone H3-labeled precursors and *olig2*-positive cells is observed in the pMN domain, from which KA’ cells are produced, suggesting a failure of KA’ cells in terminal differentiation. Although *gata2/3* and *tal1/2* encode highly related proteins and act via both the distinct and redundant functions in the central nervous system and during hematopoietic development, it is not known how these genes are functional as selector genes and/or activators for exiting the cell cycle.

## Perspectives

We currently know that *gata2/3* and *tal1/2* are critical for specifying KA’ and KA” cells, respectively, but the crucial details remain unknown as to how these two subgroups of KA cells that originated from two different progenitor domains are encoded at the genomic, epigenomic, and transcriptomic levels via transcription factors, particularly in terms of KA/CSF-cN cells that are present in all vertebrate species that have been studied. Based on a mechanistic understanding of this regulatory network, transient expression of ASCL1 and DLX2 is sufficient to convert human pluripotent stem cells exclusively into GABAergic neurons with characteristics of forebrain GABAergic neurons. Remarkably, a combination of *Ascl1* and *Dlx2* with other transcription factors, including *Arx*, *Brn4*, *Ebf1*, *Gata2*, *Gbx1*, *Gsx2*, *Ikaros*, *Islet1*, *Lhx6*, *Lmo2*, *Lmo3*, *Meis1*, *Meis2*, *Oct6*, *Otp*, *Pbx1*, and *Ptf1a* does not drive the cells to generate the different subtypes of GABAergic neurons (Yang et al., 2017), suggesting that much remains unknown regarding how these GABAergic cells are differentiated and specified. It has been indicated that regulatory elements as binding hubs are critical for regulating spatiotemporal gene expression patterns and cell lineage specifications. Although cis-regulatory control of gene expression is a complex process, dependent on distal sequences, spatial organization of the chromosome, and chromatin or epigenetic states and advances in genetics, genomics, and developmental neurobiology have helped to gain further insight into the genetically encoded wiring diagram that ultimately gives rise to KA/CSF-cN cells. In particular, single-cell RNA-sequencing methods have been demonstrated in characterizing cellular diversity and transcriptional regulation of the brain and spinal cord, shedding the new light on revealing the regulatory networks that specify KA/CSF-cN identities.

## Author Contributions

LY wrote the manuscript. FW prepared the figures. US read and commented on drafts of this manuscript. All authors contributed to the article and approved the submitted version.

## Conflict of Interest

The authors declare that the research was conducted in the absence of any commercial or financial relationships that could be construed as a potential conflict of interest.
